# Guanfacine treatment improves ADHD phenotypes of impulsivity and hyperactivity in a neurofibromatosis type 1 mouse model

**DOI:** 10.1186/s11689-019-9304-y

**Published:** 2020-01-15

**Authors:** J. L. Lukkes, H. P. Drozd, S. D. Fitz, A. I. Molosh, D. W. Clapp, A. Shekhar

**Affiliations:** 1 Department of Psychiatry, Indiana University School of Medicine, Indianapolis, IN 46202 USA; 2 Stark Neurosciences Research Institute, Indiana University School of Medicine, 320 West 15th Street, Indianapolis, IN 46202 USA; 3 Program in Medical Neurosciences, Indiana University School of Medicine, Indianapolis, IN USA; 4 Wells Center for Pediatric Research, Department of Pediatrics, Indiana University School of Medicine, Indianapolis, IN USA; 5 Department of Microbiology and Immunology, Indiana University School of Medicine, Indianapolis, IN USA; 6 Department of Pharmacology and Toxicology, Indiana University School of Medicine, Indianapolis, IN USA; 7 Indiana Clinical and Translation Sciences Institute, Indiana University School of Medicine, Indianapolis, IN USA

**Keywords:** Impulsivity, ADHD, Guanfacine, Mouse, Neurofibromatosis type 1, Delay discounting, Behavioral inhibition, Alpha 2A-adrenergic receptor agonist, Hyperactivity, Cliff avoidance reaction test

## Abstract

**Background:**

Neurofibromatosis type 1 (NF1) is an autosomal dominant disorder with a mutation in one copy of the neurofibromin gene (*NF1*^*+/−*^). Even though approximately 40–60% of children with NF1 meet the criteria for attention deficit hyperactivity disorder (ADHD), very few preclinical studies, if any, have investigated alterations in impulsivity and risk-taking behavior. Mice with deletion of a single NF1 gene (*Nf1*^*+/−*^) recapitulate many of the phenotypes of NF1 patients.

**Methods:**

We compared wild-type (WT) and *Nf1*^*+/−*^ mouse strains to investigate differences in impulsivity and hyperactivity using the delay discounting task (DDT), cliff avoidance reaction (CAR) test, and open field. We also investigated whether treatment with the clinically effective alpha-2A adrenergic receptor agonist, guanfacine (0.3 mg/kg, i.p.), would reverse deficits observed in behavioral inhibition.

**Results:**

*Nf1*^*+/−*^ mice chose a higher percentage of smaller rewards when both 10- and 20-s delays were administered compared to WT mice, suggesting *Nf1*^*+/−*^ mice are more impulsive. When treated with guanfacine (0.3 mg/kg, i.p.), *Nf1*^*+/−*^ mice exhibited decreased impulsive choice by waiting for the larger, delayed reward. *Nf1*^*+/−*^ mice also exhibited deficits in behavioral inhibition compared to WT mice in the CAR test by repetitively entering the outer edge of the platform where they risk falling. Treatment with guanfacine ameliorated these deficits. In addition, *Nf1*^*+/−*^ mice exhibited hyperactivity as increased distance was traveled compared to WT controls in the open field. This hyperactivity in *Nf1*^*+/−*^ mice was reduced with guanfacine pre-treatment.

**Conclusions:**

Overall, our study confirms that *Nf1*^*+/−*^ mice exhibit deficits in behavioral inhibition in multiple contexts, a key feature of ADHD, and can be used as a model system to identify alterations in neural circuitry associated with symptoms of ADHD in children with NF1.

## Background

Neurofibromatosis type 1 (NF1) is an autosomal dominant disorder with a mutation in one copy of the neurofibromin gene (*Nf1*^*+/−*^) and has a prevalence of approximately 1 in 3000 individuals [1–9]. *NF1* encodes neurofibromin, a well-known GTPase-activating protein (GAP) that negatively regulates p21^ras^ (RAS) activity [10–16]. *NF1*-mediated RAS pathophysiology in the central nervous system plays a role in cognitive behaviors [3, 17–20]. Hyperactivation of the RAS pathway in *Nf1*^*+/−*^ mice is known to lead to various disruptions of neurotransmitter systems and synaptic plasticity that may underlie deficits observed in cognitive behaviors related to ADHD. The clinical presentations in NF1 patients are highly variable in severity with about 20 to 40% developing benign tumors [21]. Furthermore, individuals with NF1 suffer from significant incidence of cognitive difficulties and are frequently diagnosed with learning disabilities, attention deficit hyperactivity disorder (ADHD), and autism spectrum disorders [22–25]. Even though approximately 40–60% [5] of children with NF1 meet the criteria for ADHD, very few preclinical studies have investigated the role of *NF1* mutation on ADHD phenotypes.

ADHD is one of the most frequently diagnosed neurodevelopmental disorders with an occurrence of approximately 5% in children worldwide [26–28]. The DSM-5 classifies ADHD as a patient exhibiting a persistent pattern of inattention and/or hyperactivity–impulsivity that interferes with functioning or development (American Psychiatric Association. Diagnostic and Statistical Manual of Mental Disorders, Fifth Edition. 2013). Several aspects of attention (intensive, selective, and executive) are impaired in NF1 patients with ADHD [29]. In addition, a common manifestation of ADHD in NF1 patients is executive dysfunction including impairments in response inhibition [2, 30–32]. ADHD symptoms have a negative impact on the intellectual development of children with NF1 [33]. Individuals with NF1 diagnosed with ADHD also have decreased alertness, reduced visuospatial skills, and impaired cognitive flexibility compared to healthy controls [29]. For instance, in a classic stop signal response inhibition test, during the stop signal task, NF1 patients with or without ADHD needed a significantly longer stop signal delay in order to inhibit their pre-potent response [34]. In studies, which do not stratify NF1 patients based on an ADHD diagnosis but measure ADHD phenotypes, NF1 patients exhibit greater incidence of impulsivity and hyperactivity [19, 35]. When tested for impulsivity, individuals with NF1 commit more errors and respond faster than control participants in the Go/No-Go task [35]. Indeed, NF1 children react to a target appearance more quickly (short reaction time) and commit more errors compared to control children suggesting increased impulsivity and inattention [2]. Finally, NF1 children have worse outcomes compared to control children on tasks of spontaneous and reactive cognitive flexibility [36]. These studies reflect the significant impact of ADHD in NF1 patients.

Using NF1 mutant strains of mice, investigators have been able to observe cognitive and behavioral deficits like those commonly seen in children diagnosed with NF1 [5, 20, 37]. Our previous studies have found that *Nf1*^*+/−*^ mice exhibit deficits in long-term social learning in the three-chamber preference test [20]. Furthermore, we have found that *Nf1*^*+/−*^ mice have reduced neurofibromin levels and RAS-MAPK/ERK hyperactivation in many key brain regions as well as disrupted amygdala synaptic plasticity [20]. Using *Nf1*^*+/−*^ mice that have inactivation of the neurofibromin gene within astroglial cells (specifically GFAP+ cells), known as *Nf1*^*+/−*^ *OPG* mice, Brown and colleagues found impairments in spatial learning and memory [38]. This group also observed that *Nf1*^*+/−*^ *OPG* mice have deficits in attention that can be restored by administration of methylphenidate or l-3,4-dihydroxyphenylalanine, but these mice do not display hyperactivity [38]. Previous studies have also found that *Nf1*^*+/−*^ male mice exhibit deficient pre-pulse inhibition (PPI), a deficit observed in children with ADHD [39, 40]. Using *Nf1*^*+/−*^ male mice provides strong construct validity for this autosomal dominant disorder. This study will be the first preclinical study to investigate deficits in behavioral inhibition and impulsivity in a NF1 preclinical model.

Impulsive choice has significant and enduring consequences for individuals with ADHD, and thus, effective treatment for impulsivity is of highest importance. Methylphenidate (MPH) and other stimulant medications are commonly used to treat short-term impulsivity in ADHD individuals. However, these medications work in some, but not all children with NF1 diagnosed with ADHD. Also, due to side effects and abuse potential of stimulant medications, there are additional factors that contribute to the need for alternative approaches to treating these subjects with agents such as guanfacine. Furthermore, Omrani et al. found that preclinical models of NF1 have alterations in hyperpolarization-activated cyclic nucleotide-gated channel 1 (HCN) channels, suggesting guanfacine may be particularly effective in treating ADHD in NF1 due to its effects on HCN channels in the prefrontal cortex [41]. Therefore, the current study tested the effects of guanfacine (an alpha-2a (α_2A_) adrenergic receptor agonist), which is a non-stimulant used to treat ADHD patients, in a clinically relevant experimental model of NF1. Overall, these studies may help to elucidate the molecular mechanisms of impulsivity and inhibition that can be used to develop better treatments and diagnostics for patients with NF1.

## Methods

### Animals

All experiments were conducted using adult WT and *Nf1*^*+/−*^ male mice (3–4 months old) that were bred on a C57BL/6J background for over 10 generations. Each cohort was made up of littermates with 2–4 litters used per experimental group. The original *Nf1*^*+/−*^ mice were obtained from Tyler Jacks at the Massachusetts Institute of Technology (Cambridge, MA). The breeding scheme for these mice consisted of trios with one WT male on a C57BL/6J background and two *Nf1*^*+/−*^ females on C57BL/6J background. Once weaned (postnatal day 28), all mice were group-housed (3–4/cage by litter containing mixed genotypes), given food and water ad libitum, and maintained on a 12-h light-dark cycle (7:00 am/7:00 pm) at 72 °F. Each behavioral task was done in separate cohorts of animals. Only males were used in these studies because in the general population, males are at a 3:1 higher risk to develop ADHD compared to females. Furthermore, we have observed very few phenotypic differences in *Nf1*^*+/−*^ females compared to WT controls. However, future experiments will examine ADHD-like phenotypic behavior in female *Nf1*^*+/−*^ mice. Animal care procedures were conducted in accordance with the NIH Guidelines for the Care and Use of Laboratory Animals (NIH Publication No. 80-23) revised 1996. All procedures have been approved by the Indiana University School of Medicine Institutional Animal Care and Use Committee (Protocol No. 11082).

### Drugs

Guanfacine (GUAN, 0.1 and 0.3 mg/kg, i.p [42–45]) was obtained from Sigma (St. Louis, MO), dissolved in 0.9% saline (vehicle, VEH), and administered in a volume of 1 mL/kg. Previous literature has found that a range of 0.1 to 0.3 mg/kg is an effective dose of GUAN to reduce locomotion and measures of impulsivity in rodents that are not confounded by sickness and significant sedation [44, 45]. Therefore, we used a different cohort of mice for each behavioral task and investigated the effects of two doses of GUAN (0.1 and 0.3 mg/kg) that are based on the literature. These doses of GUAN in mice are comparable to the doses used for children with ADHD ([46]; FDA ref# 3335794). To ensure observers were blind to treatment conditions, vials were letter-coded prior to administration by a person not involved in the running of each experiment.

### Behavioral tests

#### Open field test

For Experiment 1, mice (*n* = 7–8/genotype) were placed into the center of a square open field arena (40 × 40 cm, 30 cm height) [20]. The behavior of each mouse in the open field arena was recorded for 60 min on video and scored afterwards using the AnyMaze automated software (AnyMaze, Stoelting, Wood Dale, IL). To ensure observers were blind to genotype conditions, video files were letter coded prior to behavioral scoring. The total distance traveled (m) was scored. Behavior was analyzed in 10-min increments as well as the entire 60-min testing period. For Experiment 2, VEH (*n* = 8/genotype), 0.1 mg/kg GUAN (*n* = 6–8/genotype), and 0.3 mg/kg GUAN (*n* = 9/genotype) were administered to separate subgroups of mice daily for 5 days and then 30 min prior to open field testing. Due to technical difficulties with some of the videos, the total animal numbers in the open field were reduced from 9 to 8 in both WT and *Nf1*^*+/−*^ genotypes in the VEH treatment groups and reduced from 9 to 6 in the WT 0.1 mg/kg GUAN group and 8 in the *Nf1*^*+/−*^ 0.1 mg/kg GUAN group.

#### Cliff avoidance reaction (CAR)

A separate set of mice were used in the cliff avoidance reaction (CAR) test. Methods for the CAR were based on those described by Yamashita et al. [47]. For Experiment 1, a round, plastic platform (diameter, 20 cm; thickness, 2 cm) supported by a plastic rod (height, 50 cm) was used to assess CAR. The platform was stabilized by a rectangular piece of plastic (26 cm × 38 cm) and set in a kiddie pool with a rubber bottom to help prevent injury if the animal fell. At the beginning of the test, mice (*n* = 6–7/genotype) were gently placed in the center of the platform and behavior was recorded for 60 min. If a mouse fell from the platform, it was immediately placed back on the platform and the test is continued until 60 min had elapsed. A mouse was considered to have impaired CAR if it fell from the platform. CAR was calculated as % of mice which demonstrated intact CAR for each group: % of mice with intact CAR = [the number of mice that did not fall from the platform/total number of mice] × 100. Total distance traveled, number of entries into the edge zone, and number of entries into the over the edge zone were recorded and scored with the automated AnyMaze software. The edge zone was defined by outlining the outer 1 inch of the round platform with one inner circle and one outer circle in the AnyMaze software. The over the edge zone was defined by outlining an outer circle that was 2 inches from the edge of the platform in the AnyMaze software. During the testing period, ataxia and stereotypy were assessed and manually scored by an observer blind to the treatment conditions. To ensure observers were blind to genotype conditions, video files were letter-coded prior to behavioral scoring. For Experiment 2, VEH (*n* = 8–9/genotype), 0.1 mg/kg GUAN (*n* = 9/genotype), and 0.3 mg/kg GUAN (*n* = 9/genotype) were administered to a separate group of mice daily for 5 days and then 30 min prior to CAR testing.

#### Measurement of ataxia and stereotypy

At the 30-min time point during the CAR test session in Experiment 1, both ataxia and stereotypy behavior were assessed for 1 min. We assessed ataxic and stereotypy behavior during the CAR test to replicate methods from Yamashita et al [47]. Ataxic behavior was scored using the same numerical rating scale that was developed by Hiramatsu and colleagues in 1989; the following numbers were used according to the behavior [47, 48]: “(0) inactive or coordinated movements, (1) awkward or jerky movements or loss of balance while rearing, (2) frequent falling or partial impairment of reflexes, (3) inability to move beyond a small area and support of body weight on haunches or abdomen, and (4) inability to move except for twitching movements”. For stereotypy scored from video images by an observer blinded to the treatment conditions, we used a rating scale used by Creese and Iversen [49] and Yamashita et al. [47]: “(0) asleep or stationary, (1) active, (2) predominantly active but with bursts of stereotypic sniffing or rearing, (3) stereotypic activity such as sniffing along a fixed path on the test ground, (4) stereotypic sniffing or rearing maintained in one location, (5) stereotypy in one location with bursts of gnawing or licking, and (6) continual gnawing or licking”. For Experiment 2, VEH (*n* = 8–9/genotype), 0.1 mg/kg GUAN (*n* = 9/genotype), and 0.3 mg/kg GUAN (*n* = 9/genotype) were administered daily for 5 days and then 30 min prior to CAR testing.

#### Delay discounting test

Methods for the delay discounting test (DDT) were adapted from the protocol described from Freund and colleagues [50] and are illustrated in Fig. 3a. For Experiment 1, a separate set of animals (*n* = 6–8/genotype) were trained to run down an arm of a t-maze (each arm measured 36 cm length, 8 cm width, and 12.5 cm height; the entire apparatus elevated 6 cm) to receive either a small reward (1 Cocoa Krispies cereal) in one arm or a large reward (4 Cocoa Krispies) in the other arm. The number of days it took for the animal to learn to choose the large reward over the small reward was recorded. Animals were trained until they met a set criterion of choosing the large reward at least five out of six trials on two consecutive days (a total of 12 rewards maximum). During training, after the mouse makes a choice of an arm, a clear barrier is lowered so that the mouse remains in that arm while eating the chosen reward. Once subjects reached this criterion, a delay period of 10 or 20 s was initiated for the large reward while the small reward was available immediately. This testing period lasted 6 days. The number of testing days were based on the methods from Olmstead et al. [51], and other laboratories have shown this amount of testing days to sufficiently detect group differences [52]. Based on previous literature, different groups of animals were used for each delay condition to ensure there were no carryover effects between delay conditions [50]. The 0 delay measure is when the animals meet the criteria of choosing the large reward 10 out of 12 trials across two days and, therefore, refers to the number of small rewards chosen on day 1 and day 2 prior to the testing period. For the 6 days of testing, the data from the last 2 days (day 5 and day 6) is used to determine the effect of genotype and/or treatment on impulsivity. The number of times the small reward is chosen on day 5 and day 6, out of 12 total trials, is compared. To ensure observers were blind to genotype conditions, animal cages were letter-coded prior to behavioral testing. For Experiment 2, VEH (*n* = 6/genotype) and 0.3 mg/kg GUAN (*n* = 6/genotype) were administered to a separate cohort of DDT trained WT and *Nf1*^*+/−*^ mice immediately prior to the start of each testing day (across 6 days in total). Since DDT training and testing can take up to 4 weeks, we chose to use only the 0.3 mg/kg GUAN dose for this test. All data were recorded by an observer blind to the treatment conditions.

### Statistical analysis

Open field data were analyzed using a two-way ANOVA with repeated measures with *genotype* as the main factor and *time* as repeated measures. Total distance traveled during the entire test period in the open field test were expressed as mean + SEM and analyzed with a Student’s *t* test. In the CAR test, a Student’s *t* test was used to assess the effect of genotype on the distance traveled during the CAR test as well as stereotypy and ataxic scores. Furthermore, a Chi square test was used to assess the effect of genotype on the percentage of mice that displayed CAR impairment. A two-way ANOVA with main effects of *genotype* and *location* was used to analyze the number of entries into different locations on the CAR platform. Delay discounting data were analyzed using a two-way ANOVA with *time of delay* and *genotype* as the main factors, and in the presence of significance, post hoc analyses were conducted with Sidak’s multiple comparisons test. The effects of GUAN on distance traveled were analyzed using a three-way ANOVA with repeated measures with *genotype* and *treatment* as the main factors and *time* as the repeated measures. A Chi square test was used to assess the effects of GUAN on the % of mice with intact CAR within each genotype, whereas total distance traveled and stereotypy scores were analyzed using a two-way ANOVA with *genotype* and *treatment* as the main factors. The effects of GUAN on delay discounting were assessed using a two-way ANOVA with *treatment* and *genotype* as the main factors. In the presence of a significant main effect, post hoc analyses for all ANOVAs were assessed with Sidak’s multiple comparisons test. Statistical significance was accepted with *p* ≤ 0.05. All statistical analyses were carried out using GraphPad Prism 7.0 (GraphPad Software, La Jolla, CA) and SPSS 25.0 (IBM Corp.). All graphs were generated using GraphPad Prism 7.0. For a significance level of *α* = 0.05, using means and standard deviations obtained from preliminary studies, we calculated power using a power calculator available from “Online Statistics Education: An Interactive Multimedia Course of Study – XXI:9” (David M. Lane; jStat, MIT license). For n = 6, open field data resulted in 0.828 power. For n = 6, CAR data resulted in 0.894 power. For n = 6, DDT data resulted in 0.899 power. Therefore, these studies are sufficiently powered. The effect size was determined by taking the difference between the means and dividing it by the standard deviation*.* Furthermore, all data were tested for normality with a Shapiro–Wilk normality test.

## Results

### Experiment 1: Phenotyping *Nf1*^*+/−*^ mice for ADHD-associated behaviors

#### One hour open field

**Fig. 1:**
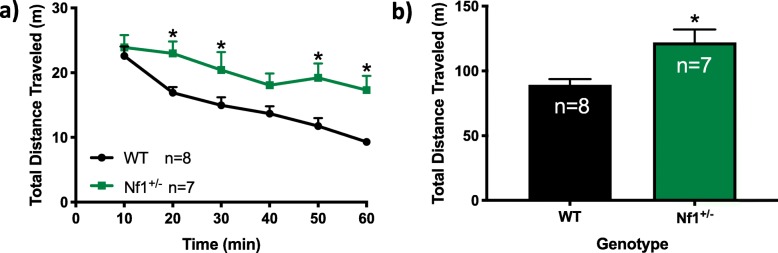
*Nf1*^*+/−*^ mice exhibit hyperactivity in response to a novel open field. Graphs depict the differences in behaviors between WT and *Nf1*^*+/−*^ mice on the **a** distance (m) traveled over time, and the **b** total distance moved (m) in 60 min. *p < 0.05 compared to wild-type (WT). All data reported as mean + S.E.M

*Nf1*^*+/−*^ mice exhibit increased distance traveled during the 60-min testing period compared to WT mice (Fig. 1a, b). A two-way ANOVA with repeated measures revealed main effects of time (*F*_5,65_ = 17.51, *p* < 0.0001) and genotype (*F*_1,13_ = 9.707, *p* = 0.0082) without a time × genotype interaction (*F*_5,65_ = 1.996, *p* = 0.0908). Post hoc analyses of between subject effects at every 10-min time segment demonstrated that *Nf1*^*+/−*^ mice exhibit increased total distance traveled at 20 min (*p* = 0.0113), 30 min (*p* = 0.0224), 50 min (*p* = 0.0021), and 60 min time points (*p* = 0.0010; Fig. 1a). Overall, *Nf1*^*+/−*^ mice exhibit an increased amount of total distance traveled within the total 60-min testing period (*t* = 3.116, *df* = 13, *p* = 0.0082; Fig. 1b).

#### Cliff avoidance reaction (CAR) test

**Fig. 2:**
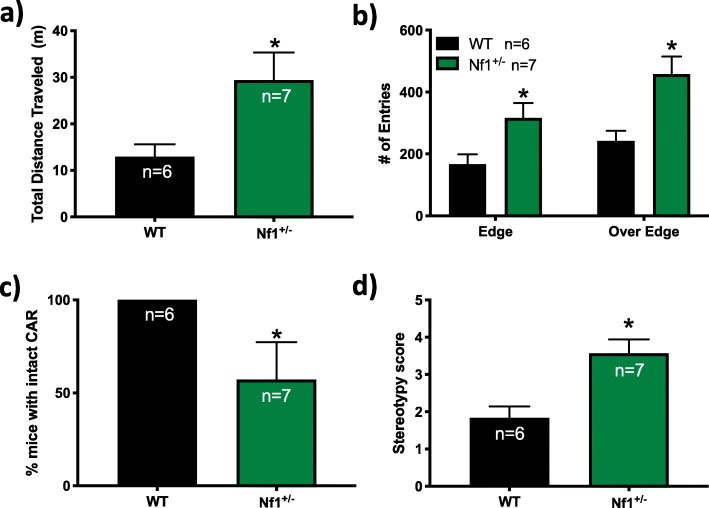
*Nf1*^*+/−*^ mice exhibit deficits in behavioral inhibition in the cliff avoidance reaction (CAR) test. Graphs illustrate the effects of *Nf1*^*+/−*^ on the **a** total distance traveled (m), **b** number of entries made into the edge zone and outer edge zone of the CAR platform, **c** % mice with intact CAR, and **d** stereotypy rating scores. *p < 0.05 compared to wild-type (WT). All data reported as mean + S.E.M

*Nf1*^*+/−*^ mice had an increased amount of total distance traveled during the 60-min CAR test compared to WT mice (*t* = 2.529, *df* = 10, *p* = 0.0299), once again suggesting hyperactivity in *Nf1*^*+/−*^ mice (Fig. 2a). Comparing the number of entries into the center and edges of the platform, a two-way ANOVA found main effects of location (*F*_1,22_ = 5.672, *p* = 0.0263) and genotype (*F*_1,22_ = 16.3, *p* = 0.0006), but not an interaction between location and genotype (*F*_1,22_ = 0.5208, *p* = 0.4781). *Nf1*^*+/−*^ mice exhibited an increased number of entries (*p* = 0.0285) into the edge zone of the platform compared to WT (*p* = 0.2702; Fig. 2b). In addition, *Nf1*^*+/−*^ mice placed a large portion of their heads and torsos over its edge and made an increased amount of entries into the over the edge zone where they attempted to climb underneath the platform (*p* = 0.0028) compared to WT controls (*p* = 0.0324; Fig. 2b). These data suggest that *Nf1*^*+/−*^ mice display increased deficits in behavioral inhibition, and it was common for *Nf1*^*+/−*^ mice to fall off the platform. Falling at least once from the platform is referred to as impaired CAR. Over half of the *Nf1*^*+/−*^ mice exhibited impaired CAR during the 60-min test (Fig. 2c). However, no WT mice displayed impaired CAR (Fig. 2c). This was confirmed by a significant difference of % mice with intact CAR between WT and *Nf1*^*+/−*^ mice (Chi square = 54.78 (1), *p* < 0.0001). In addition, stereotypy scores of *Nf1*^*+/−*^ mice were significantly higher than those of WT mice (*t* = 3.544, *df* = 11, *p* = 0.0046; Fig. 2d). However, neither WT nor *Nf1*^*+/−*^ mice showed any significant signs of ataxic behavior (data not shown; all animals regardless of genotype received a 0 score). These data suggest an impulsive phenotype in *Nf1*^*+/−*^ mice.

#### Delay discounting test

**Fig. 3:**
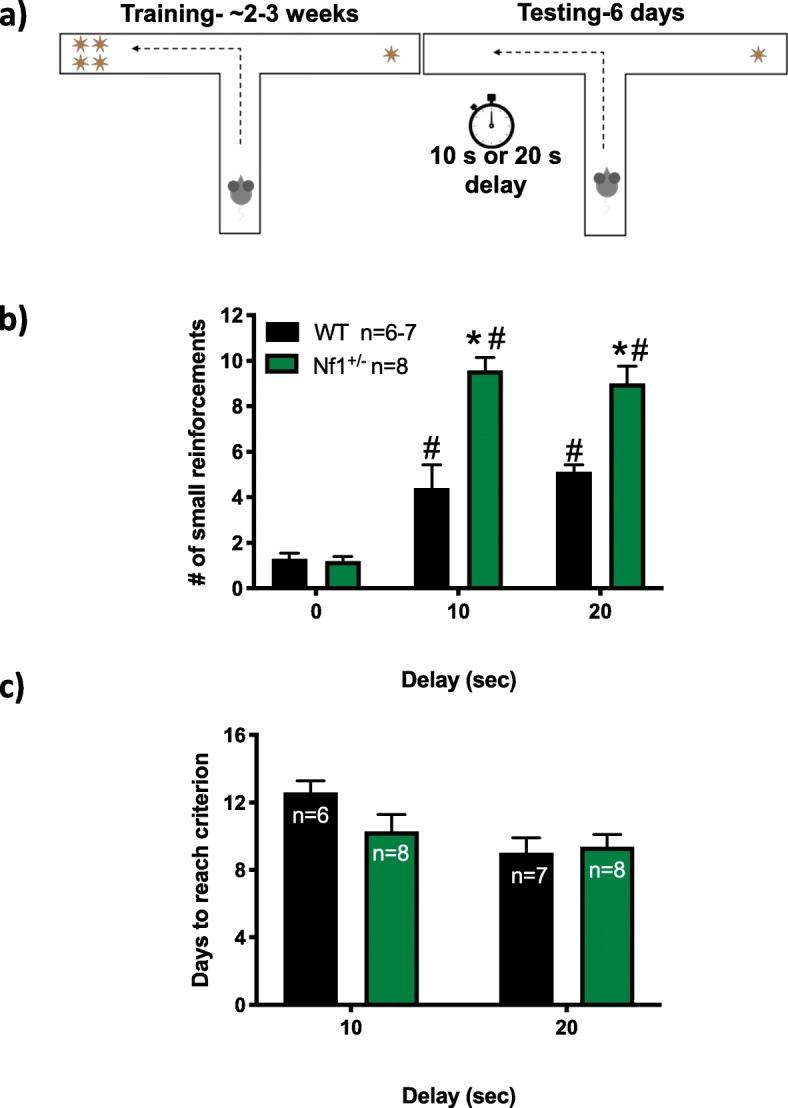
*Nf1*^*+/−*^ mice exhibit increased impulsive choice in the delay discounting test (DDT). Graphs illustrate **a** the general methods used in the DDT on a t-maze and the effects of *Nf1*^*+/−*^ on **b** the number of small reinforcements chosen during a DDT task in a t-maze and **c** the number of days the mice took to reach criterion prior to the initiation of the delay. *p < 0.05 compared to wild-type (WT). #*p* < 0.05 compared to 0 delay within the same genotype. All data reported as mean + S.E.M

In the delayed discounting test (Fig. 3), a two-way ANOVA revealed main effects of delay (*F*_2,50_ = 130.2, *p* < 0.0001) and genotype (*F*_1,50_ = 60.98, *p* < 0.0001) as well as an interaction between delay and genotype (*F*_2,50_ = 20.8, *p* < 0.0001). Post hoc analyses suggest that upon delayed initiation, both WT and *Nf1*^*+/−*^ mice selected the smaller reward more frequently (*p* < 0.0001; Fig. 3b). *Nf1*^*+/−*^ mice chose an increased number of smaller reinforcements at both the 10 and 20 s delay compared to WT controls (*p* < 0.0001), suggesting increased impulsivity in *Nf1*^*+/−*^ mice.

No significant differences were found for the number of days required to reach criterion because of genotype (*F*_1,24_ = 1.185, *p* = 0.2872; Fig. 3c). There was a main effect of delay (*F*_1,24_ = 6.41, *p* = 0.0183) but no interaction between delay and genotype (*F*_1.24_ = 2.278, *p* = 0.1442). On average, *Nf1*^*+/−*^ mice required 10.29 ± 1.02 days to reach criterion, whereas WT mice required 12.60 ± 0.68 days for the 10 s delay (Fig. 3c). For the 20 s delay test, *Nf1*^*+/−*^ mice required 9.38 ± 0.84 days to reach criterion, whereas WT mice required 9.00 ± 0.82 days. These data suggest that the *Nf1* mutation did not produce learning or motivational deficits in this reward-driven behavioral test.

### Experiment 2: Determining the effects of GUAN on ADHD-associated behaviors in *Nf1*^*+/−*^ mice

#### One hour open field with GUAN administration

**Fig. 4:**
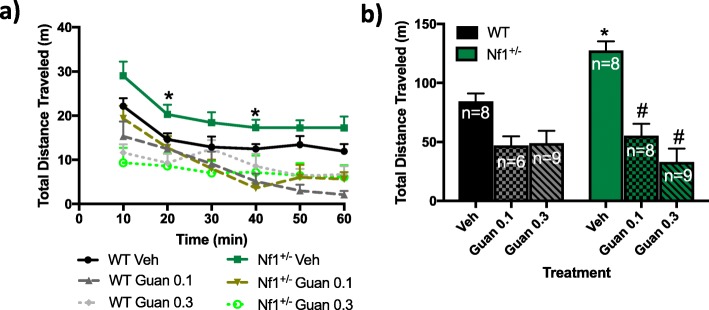
Guanfacine (GUAN) decreases hyperactivity in *Nf1*^*+/−*^ mice. Graphs depict the effects of GUAN (0.1 and 0.3 mg/kg) on the **a** amount of distance (m) traveled over time and the **b** total distance moved (m) in 60 min. *p < 0.05 compared to wild-type (WT) within the same treatment. #*p* < 0.05 compared to vehicle (VEH) within the same genotype. All data reported as mean + S.E.M

*Nf1*^*+/−*^ mice treated with VEH exhibit increased distance traveled in response to a novel open field compared to WT (Fig. 4a, b). A three-way ANOVA with repeated measures revealed main effects of time (*F*_5,195_ = 28.750, *p* < 0.0001) as well as a time × treatment interaction (*F*_5,195_ = 4.150, *p* < 0.0001). However, no main effects of genotype nor other interactions were observed (all *p* > 0.05). In VEH-treated animals, post hoc analyses revealed that *Nf1*^*+/−*^ mice exhibit increased total distance traveled at 20 and 40 min compared to WT mice (*p* < 0.05; Fig. 4a). Administration of 0.1 and 0.3 mg/kg GUAN over 6 days decreased the total distance moved over time in both *Nf1*^*+/−*^ and WT mice (*p* < 0.05; Fig. 4a). A two-way ANOVA revealed main effects of treatment (*F*_2,41_ = 51.58, *p* < 0.0001) as well as a genotype × treatment interaction (*F*_2,41_ = 9.525, *p* = 0.0098), but no main effects of genotype (*F*_1,41_ = 2.106, *p* = 0. 1375) in the open field test for total distance moved during the 60-min testing period (Fig. 4b).

#### CAR test with GUAN administration

**Fig. 5:**
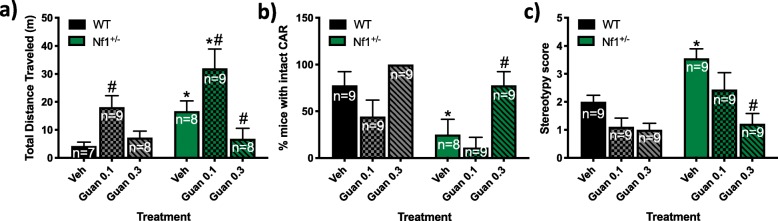
Guanfacine (GUAN, 0.3 mg/kg) attenuates deficits in behavioral inhibition in *Nf1*^*+/−*^ mice in the cliff avoidance reaction (CAR) test. Graphs illustrate the effects of *Nf1*^*+/−*^ on the **a** total distance traveled (m), **b** % mice with impaired CAR, and **c** stereotypy rating scores. *p < 0.05 compared to wild-type (WT) within the same treatment. #*p* < 0.05 compared to vehicle (VEH) within the same genotype. All data reported as mean + S.E.M

Administration of 0.3 mg/kg GUAN for 6 days prior to the CAR test attenuated impairments in behavioral inhibition observed in *Nf1*^*+/−*^ mice throughout the test (Fig. 5). A two-way ANOVA found main effects of treatment (*F*_2,42_ = 27.56, *p* = 0.0003) and genotype (*F*_1,42_ = 9.917, *p* = 0.0114), but not an interaction between treatment × genotype (*F*_2,42_ = 4.006, *p* = 0.2544) in the CAR test on total distance traveled during the 60-min testing period. *Nf1*^*+/−*^ mice exhibited increased total distance traveled following VEH administration compared to VEH-treated WT mice (*p* = 0.018; Fig. 5a). Administration of 0.3 mg/kg GUAN attenuated the higher total distance traveled observed in *Nf1*^*+/−*^ mice (*p* < 0.021) but had no effect on WT mice (*p* > 0.9999; Fig. 5a). In contrast, administration of 0.1 mg/kg GUAN increased distance traveled in *Nf1*^*+/−*^ mice compared to WT (*p* = 0.0285). More than half of the *Nf1*^*+/−*^ mice exhibited impaired CAR during the 60-min test (Fig. 5b). Compared to VEH-treated WT, VEH-treated *Nf1*^*+/−*^ mice more frequently exhibited impaired CAR (*p* < 0.0001; Fig. 5b). Administration of GUAN did have dose-dependent effects on CAR performance (Chi square = 18.99 (2), *p* < 0.0001). Administration of 0.3 mg/kg GUAN but not 0.1 mg/kg GUAN improved impaired CAR in *Nf1*^*+/−*^ mice (*p* < 0.05; Fig. 5b). In addition, a two-way ANOVA found main effects of treatment (*F*_2,48_ = 24.52, *p* = 0.0002) and genotype (*F*_1,48_ = 14.05, *p* = 0.0012) on stereotypy score, with a non-significant treatment and genotype interaction (*F*_2,48_ = 4.444, *p* = 0.1649) on stereotypy score (Fig. 5c). Post hoc tests revealed that 0.3 mg/kg GUAN administration decreased stereotypy score in *Nf1*^*+/−*^ mice (*p* = 0.0007; Fig. 5c). No differences were observed in ataxic behavior regardless of genotype and treatment (data not shown; all animals received a 0 score).

#### Delay discounting with GUAN administration

**Fig. 6:**
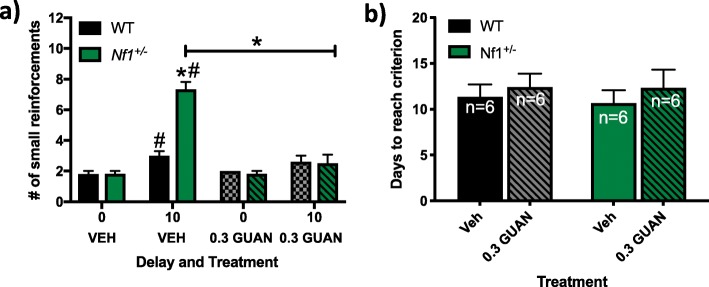
Guanfacine (GUAN, 0.3 mg/kg) decreases impulsive choice in *Nf1*^*+/−*^ mice in the delay discounting test (DDT). Graphs depict the effects of GUAN on **a** the number of small reinforcements chosen during a DDT task in a t-maze and **b** the number of days the mice took to reach criterion prior to the initiation of the delay. *p < 0.05 compared to wild-type (WT) within the same treatment. #*p* < 0.05 compared to vehicle (VEH) within the same genotype. All data reported as mean + S.E.M. Black-filled bars represent WT mice treated with VEH. Green-filled bars represent *Nf1*^*+/−*^ mice treated with VEH. Gray-filled bars with pattern represent WT mice treated with GUAN. Green-filled bars with pattern represent *Nf1*^*+/−*^ mice treated with GUAN

Administration of GUAN to *Nf1*^*+/−*^ mice attenuated the amount of delay discounting to WT control levels (Fig. 6). Since *Nf1*^*+/−*^ mice exhibited similar behavior in response to both the 10- and 20-s delays, we chose to only investigate the effects of GUAN administration on impulsive choice in response to the 10-s delay (Fig. 3b). A two-way ANOVA revealed main effects of treatment (*F*_3,36_ = 39.51, *p* < 0.0001) and genotype (*F*_1,36_ = 16.84, *p* < 0.0001) as well as an interaction between treatment and genotype (*F*_3,36_ = 19.52, *p* < 0.0001). Post hoc analyses suggest that upon initiation of a 10-s delay, both VEH-treated WT and *Nf1*^*+/−*^ mice selected the smaller reward more frequently (*p* < 0.0001; Fig. 6a). However, *Nf1*^*+/−*^ mice treated with VEH chose an increased number of smaller reinforcements in response to the 10-s delay compared to WT controls (*p* < 0.0001; Fig. 6a). These data confirm that the *Nf1*^*+/−*^ mutation leads to increased impulsivity. GUAN treatment decreased the number of small reinforcements *Nf1*^*+/−*^ mice chose in response to the 10-s delay compared to VEH-treated *Nf1*^*+/−*^ mice (*p* < 0.05; Fig. 6a).

No significant differences were found for the number of days required to reach criterion as a result of genotype (*F*_1,19_ = 0.05266, *p* = 0.8210) and treatment (*F*_1,19_ = 0.7316, *p* = 0.4030; Fig. 6b). Furthermore, there was not a significant interaction between treatment and genotype (*F*_1,19_ = 03525, *p* = 0.8531). These data suggest that the *Nf1*^*+/−*^ mutation did not produce learning or motivational deficits in this reward-based behavioral test.

## Discussion

A high percentage of NF1 children are diagnosed with ADHD [3, 53]. Furthermore, NF1 children tend to be impulsive and struggle with social cues, which can lead to poor scholastic performance [54–57]. In this study, we report for the first time that mutation of the NF1 gene in male mice leads to hyperactivity, impulsivity, and deficits in behavioral inhibition. We also show that treatment with a commonly used non-stimulant ADHD medication, GUAN, attenuates these deficits in *Nf1*^*+/−*^ mice.

*Nf1*^*+/−*^ mice exhibited increased distance traveled compared to WT controls in a 1-hr open field test. These data suggest that *Nf1*^*+/−*^ mice exhibit hyperactivity in an open field compared to WT mice. Furthermore, *Nf1*^*+/−*^ mice may also be displaying a lack of habituation to the novel open field environment since a similar level of locomotion compared to WT mice was observed in the first 10 min of the open field test. In contrast to our current findings, previous studies have found that *Nf1*^*+/−*^ OPG mice display decreased total ambulation and reduced exploratory behavior in a novel environment [38]. In general, these data suggest that both *Nf1*^*+/−*^ and *Nf1*^*+/−*^ OPG mice display abnormal locomotor activity. Differences in locomotor activity may be explained by the unique genetic engineering of the models. While *Nf1*^*+/−*^ mice used in this study are haploinsufficient in all cells, *Nf1*^*+/−*^ OPG mice are haploinsufficient in all cells except for GFAP+ cells that are *Nf1*^*−/−*^. Due to the role that glial cells play in neurodevelopment and maintenance, it can be expected that there would be some behavioral differences in these models. In particular, Brown et al. [38] suggest that *Nf1*^*+/−*^ neurons and *Nf1*^*−/−*^ glial cells both contribute to dopamine (DA) homeostasis, so we have two distinctive models to study the role of DA and norepinephrine (NE) in NF1 and how the interaction of glial cells contribute to the regulation of these neural systems and resulting behaviors. *Nf1*^*+/−*^ OPG mice provide the unique experimental model of studying the role of astrocytes in behavior, while *Nf1*^*+/−*^ mice provide an experimental model for the wider NF1 clinical population. Hyperactivity is a core symptom found in patients with ADHD as well as NF1 patients diagnosed with ADHD [19, 35]. This hyperactivity in *Nf1*^*+/−*^ mice was reduced with both 0.1 and 0.3 mg/kg GUAN administration. In general, we did observe reduced distance traveled in response to GUAN treatment regardless of genotype or dose over time. GUAN may be having biochemical effects both centrally and peripherally on the α_2A_ adrenoceptors that contribute to this observed behavior modulation [58]. The most common adverse effects reported with GUAN studies in ADHD is sedation [59], which could also be contributing to some of the reduction in distance traveled in WT mice. While sedation may be having an effect, it is well established that GUAN improves hyperactivity and attention independent of sedation.

*Nf1*^*+/−*^ mice exhibited increased hyperactivity and impulsivity compared to WT mice in the CAR test. Increased entries into the edge and outer edge zones of the platform where mice risk falling was observed more frequently in *Nf1*^*+/−*^ mice. Furthermore, *Nf1*^*+/−*^ male mice displayed impaired CAR, increased total distance moved, and high levels of stereotypical behavior during CAR testing compared to WT mice*.* It is important to note that impaired CAR observed in *Nf1*^*+/−*^ mice may be a result of their overall hyperactivity and possible lack of habituation to the novel environment. Moreover, increased stereotypic behavior, such as sniffing along a fixed path, may have brought *Nf1*^*+/−*^ mice to the edge more frequently and played a role in the observed CAR impairment. Repeated treatment with GUAN had dose-dependent effects in the CAR test. Administration of the lower 0.1 mg/kg GUAN dose increased total distance traveled in the CAR test regardless of genotype, but did not have significant effects on the % mice with intact CAR or stereotypy score. However, treatment with 0.3 mg/kg GUAN ameliorated these behavioral deficits in *Nf1*^*+/−*^ mice. Since there was not a significant effect of 0.3 mg/kg GUAN on distance traveled in WT mice during the CAR test, our data suggest that the beneficial effects of 0.3 mg/kg GUAN in *Nf1*^*+/−*^ mice may be independent from a sedative action. However, it is possible that the lower general activity observed in animals treated with 0.3 mg/kg GUAN may have led to less exploration of the outer edge, thereby decreasing the chance that the mice would fall off. Overall, these data from the CAR test recapitulate a core symptom of ADHD observed in NF1 patients: deficits in response to inhibition [2, 30, 60]. In different experimental and clinical models, GUAN improves working memory and regulates attention, cognitive performance, and behavioral inhibition [61, 62]. These data provide predictive validity for *Nf1*^*+/−*^ mice as a strong preclinical experimental model of deficits in behavioral inhibition found in NF1 patients.

Increased impulsive behavior in *Nf1*^*+/−*^ male mice was also observed in a DDT that was attenuated by 0.3 mg/kg GUAN treatment. *Nf1*^*+/−*^ mice chose a higher percentage of smaller rewards with both 10 and 20-s delays compared to WT mice, suggesting *Nf1*^*+/−*^ mice are more impulsive. When treated with GUAN (0.3 mg/kg, i.p.) daily across the 6-day testing phase of the DDT, *Nf1*^*+/−*^ mice exhibited decreased impulsive choice by waiting for the larger, delayed reward more frequently. Our data are in line with some of the clinical ADHD phenotype observed in NF1 patients. In the Go/No-Go task, a task that tests the ability to control impulsive behavior [63], individuals with NF1 commit more errors and respond faster than controls without NF1 [35]. In general, faster responses in the Go/No-Go task suggest a less cautious, more impulsive strategy that are indicative of deficits in impulse control [35]. Furthermore, reduced activation of the prefrontal cortex (PFC) during inhibition of responses during a Go/No-Go task was observed in NF1 patients [54]. Administration of GUAN moderated the influence of affective cues on response execution in the Go/No-Go task [58]. In monkeys, GUAN also promotes delay in the most important rewards as compared to immediate rewards, and improves impulse control [64]. Overall, GUAN has reduced impulsive behavior in clinical studies of ADHD and in preclinical studies of NF1; our data confirms the efficacy of this treatment for the first time in a preclinical NF1 model.

NF1 is likely to cause a variety of structural, functional, and neurochemical alterations in the central nervous system that are associated with the learning impairments observed in patients with NF1 [34, 65]. The results of the studies reported here support that *Nf1*^*+/−*^ mice are a useful experimental model for studying behavioral inhibition, and to elucidate the underlying mechanisms. For example, abnormal inhibition, especially related to GABAergic neurotransmission in NF1 patients may underlie some of their cognitive impairments associated with ADHD [35]. For instance, magnetic resonance spectroscopy found decreased GABA levels in the medial frontal cortex of NF1 patients [35]. However, a correlational analysis done in this same study suggest that high GABA was associated with a faster response style in NF1 patients in the Go/No-Go task, whereas a more cautious strategy was found in control patients [35]. In *Nf1*^*+/−*^ mice, increased inhibitory drive was observed throughout the brain [64–66]. This is consistent with numerous preclinical studies of NF1 mouse models reporting that GABA tone is notably higher in key brain regions including the prefrontal cortex, striatum, amygdala, and hippocampus [20, 22, 67–69]. This altered excitation/inhibition balance is linked to cognitive deficits in NF1 mouse models that mirror deficits observed in NF1 patients [20, 40, 68]. Therefore, aberrant GABAergic signaling may underlie the deficits in behavioral inhibition observed in the current study. How selectively modulating noradrenergic neurotransmission with GUAN regulates this inhibitory abnormality to restore function is an important objective to further elucidate the structural, functional, and neurochemical alterations occurring in NF1. Okada and colleagues have started investigating this and suggest that GUAN has dual actions on noradrenergic transmission via GABAergic disinhibition in the orbitofrontal cortex [70].

Dysfunction of the DA and/or NE nervous systems is a commonly accepted mechanism that leads to ADHD [71]. This dysfunction is also evident in NF1 models. Preclinical studies in *Nf1*^*+/−*^ *OPG* mice have found impaired DA homeostasis, such as reduced DA levels, postsynaptic DA signaling, and presynaptic DAT expression in the striatum [38]. Furthermore, DA innervation in the hippocampus was also disrupted in *Nf1*^*+/−*^ *OPG* mice as well as hippocampal D1/D5 DA receptor function [37, 72–75]. It is important to note that these alterations in DA levels were only evident in *Nf1*^*+/−*^ *OPG* mice that have inactivation of neurofibromin in GFAP+ cells [38]. The stimulant, methylphenidate (MPH), increases extracellular catecholamine levels by blocking DA and NE reuptake via the DA transporter (DAT) [76, 77] and/or NE transporters (NET) in a region-specific manner [58, 59]. Previous studies examined the effects of MPH in NF1 children with attention problems [53]. In *Nf1*^*+/−*^ *OPG* mice, treatment with MPH increased striatal DA levels, restored attention deficits, and ameliorated spatial learning deficits [38, 78]. Alterations in DA levels, signaling, and expression in the *Nf1*^*+/−*^ mouse experimental model used in the current study have yet to be investigated. Studies are currently being conducted in our laboratory to investigate dopamine and norepinephrine systems in the *Nf1*^*+/−*^ mouse model that may uncover insights into these systems and their roles in both NF1 and in ADHD.

Treatments for ADHD have mainly been focused on using psychostimulants such as MPH or amphetamine that act primarily on catecholaminergic presynaptic mechanisms [62]. However, the non-stimulant GUAN for the treatment of ADHD is also well accepted. We chose to investigate the effects of GUAN on measures of hyperactivity and impulsivity in the current study because GUAN has clinical efficacy in the treatment of ADHD, and yet, few preclinical studies have investigated its effectiveness in a preclinical model of NF1. GUAN binds α_2A_ adrenoceptors on postsynaptic dendritic spines of prefrontal pyramidal cells, strengthening prefrontal cortex (PFC) circuits [62, 66]. Specifically, at the postsynaptic level, GUAN inhibits cyclic adenosine monophosphate (cAMP) production and closes HCN and KCNQ potassium channels to enhance signals from pyramidal neurons in the PFC [79, 80]. As the PFC plays a critical role in executive function and alterations in this area underlie symptoms of ADHD, augmentation of the PFC reduces ADHD behaviors. Thus, GUAN may address two key targets: improving functionality of the PFC and influencing noradrenergic function [62, 81]. Behavioral deficits, such as increased impulsiveness, hyperactivity, and poor attention, are restored following the administration of α_2A_ adrenergic agonists, such as GUAN [63, 82]. Therefore, it can be inferred that endogenous production of NE is significant to α_2A_ receptor regulation of the PFC [62, 83]. For instance, iontophoretic delivery of GUAN into the PFC increases delay in neuronal discharges; this delay is essential for enhancing working memory [84]. Moreover, neuroimaging studies in primates and humans have observed that GUAN administration increased blood flow in the PFC as well as improved working memory, only affecting areas related to cognitive performance [85]. Future studies will need to examine catecholamine levels in *Nf1*^*+/−*^ mice and α_2A_ receptors within the PFC as well as how GUAN acts on these systems to improve ADHD-associated behaviors.

## Conclusions

Our study is the first to show that *Nf1*^*+/−*^ mice exhibit reduced behavioral inhibition leading to increased impulsivity. Furthermore, it is the first preclinical study to show that GUAN treatment can attenuate the observed impairments in *Nf1*^*+/−*^ mice. Overall, our study confirms that *Nf1*^*+/−*^ mice exhibit deficits in behavioral inhibition, a key feature of ADHD, that are amenable to pharmacological therapies used clinically for ADHD. These data suggest that *Nf1*^*+/−*^ mice are a useful experimental model to identify alterations in neural circuitry associated with core symptoms of ADHD.

## Data Availability

All data and materials used during the current study are available upon reasonable request.

## Abbreviations

ADHD: Attention deficit hyperactivity disorder
cAMP: Cyclic adenosine monophosphate
CAR: Cliff avoidance reaction
DA: Dopamine
DAT: Dopamine transporter
DDT: Delay discounting task
DSM-5: Diagnostic and Statistical Manual of Mental Disorders, fifth edition
GUAN: Guanfacine
HCN: Hyperpolarization-activated cyclic nucleotide-gated channels
MPH: Methylphenidate
NF1: Neurofibromatosis type 1
*Nf1*^*+/−*^*OPG*: Heterozygous inactivation of the mouse NF1 gene within astroglial cells
*Nf1*^*+/−*^: Heterozygous mutation in mouse NF1 gene
PFC: Prefrontal cortex
VEH: Vehicle
WT: Wild-type
α_2A_: Alpha-2A
